# Resultados da Intervenção Coronariana Percutânea Recomendada pelas Diretrizes em Mulheres com Doença Arterial Coronariana Obstrutiva: um Estudo de Coorte Longitudinal

**DOI:** 10.36660/abc.20240249

**Published:** 2025-01-22

**Authors:** Tacianne Rolemberg Braga Delamain, José Henrique Herrmann Delamain, Sergio Luiz Navarro Braga, Ricardo Costa, Dimytri Alexandre Alvim de Siqueira, Fausto Feres, Marinella Patrizia Centemero

**Affiliations:** 1 Instituto Dante Pazzanese de Cardiologia São Paulo SP Brasil Instituto Dante Pazzanese de Cardiologia, São Paulo, SP – Brasil

**Keywords:** Intervenção Coronariana Percutânea, Mulheres, Doença da Artéria Coronariana, Fatores de Risco

## Abstract

**Fundamento:**

Estudos prévios demonstram que mulheres com doença arterial coronariana (DAC) são menos submetidas a angiografia e apresentam resultados menos favoráveis após intervenção coronariana percutânea (ICP).

**Objetivos:**

Avaliar os resultados de mulheres com síndrome coronariana aguda (SCA) e DAC estável (lesão>50%) tratadas com ICP contemporânea usando stents liberadores de drogas.

**Métodos:**

Estudo de coorte observacional, longitudinal, com acompanhamento prospectivo, que incluiu todas as pacientes do sexo feminino > 18 anos admitidas em centro cardiológico público terciário no Brasil, no período de janeiro de 2019 a dezembro de 2020.

**Resultados:**

1146 mulheres (idade média de 65 anos) foram submetidas à ICP recomendada pela diretriz. Os fatores de risco foram frequentes (hipertensão: 88%, dislipidemia: 85%, diabetes: 47,5%) e 69% foram internadas devido à SCA. O acesso radial foi usado em 59% das pacientes; 1516 vasos foram tratados com 1725 stents implantados (1,5 stents/paciente). A ICP foi bem-sucedida em 97,7%, a morte intra-hospitalar ocorreu em 1,2%, IM periprocedimento em 3,6% e ataque isquêmico transitório em 0,4%. Preditores de eventos adversos cardíacos e cerebrovasculares maiores (ECCAM) intra-hospitalares: acidente vascular cerebral prévio (OR: 2,97; IC: 1,06-7,15; p = 0,023), DRC (OR: 3,11; IC: 1,49-6,20; p = 0,002) e pelo menos uma falha de procedimento durante ICP (OR: 10,2; IC: 1,17-5,9; p < 0,001). O acompanhamento médio foi de 576,2 dias em 1.047 pacientes. Mortalidade por todas as causas ocorreu em 5,3%, morte cardíaca em 3,5%, nova SCA em 8% e necessidade de nova revascularização em 5,5%. Os preditores de ECCM durante o seguimento foram admissão por SCA (retirar índice ICP) e a presença de ECCM durante a hospitalização (OR: 6,66; HR: 2,42-18,3; p< 0,001).

**Conclusão:**

Neste estudo pioneiro envolvendo 1146 pacientes tratados por ICP contemporânea e acompanhados por quase 2 anos, obtivemos resultados hospitalares e de médio prazo muito encorajadores.

## Graphical abstract

**Figura Central:**
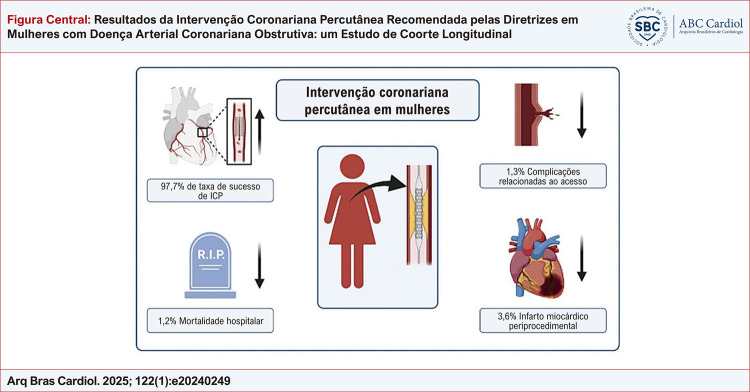
: Resultados da Intervenção Coronariana Percutânea Recomendada pelas Diretrizes em Mulheres com Doença Arterial Coronariana Obstrutiva: um Estudo de Coorte Longitudinal

## Introdução

As doenças cardiovasculares são uma ameaça significativa para as mulheres, sendo a doença arterial coronariana (DAC) o principal fator responsável por esta tendência preocupante.^1^ Embora o impacto da DAC nos homens tenha sido amplamente documentado, dados recentes ressaltam seu risco substancial para a saúde das mulheres.^2,3^ Num olhar mais atento, torna-se evidente que tanto o sexo biológico como os fatores socioculturais e de gênero desempenham um papel significativo no desenvolvimento da doença coronária e na definição do seu percurso nas mulheres. A intrincada interação entre sexo e gênero tem sido cada vez mais reconhecida devido às suas profundas implicações.^4,5^ Fatores biológicos, incluindo artérias coronárias menores e maior incidência de doença cardíaca isquêmica (DCI) não-obstrutiva, sem dúvida, contribuem para a apresentação única da DAC e, portanto, para os resultados do seu tratamento em mulheres.^6,7^ Além disso, o gênero e o aspecto sociocultural têm uma influência sutil, porém importante. Fatores como estressores sociais, barreiras ao acesso a serviços de saúde e sintomas atípicos afetam desproporcionalmente as mulheres e têm sido cada vez mais reconhecidos por seu papel no atraso do diagnóstico e do tratamento subsequente.^8,9^ Além disso, a predominância histórica de pacientes do sexo masculino em ensaios clínicos influenciou o desenvolvimento de diretrizes de tratamento, resultando no subtratamento da DCI em mulheres.^10^

Especificamente em relação à intervenção coronariana percutânea (ICP) em mulheres, piores resultados foram relatados, mesmo na era contemporânea usando stents liberadores de drogas (SLD).^11,12^ As evidências sugerem que as mulheres recebem diagnósticos e terapias recomendados pelas diretrizes com menos frequência do que os homens, potencialmente levando a um impacto significativo nos resultados após ICP.^13,14^ Pesquisas recentes demonstram que as mulheres têm uma incidência maior de eventos cardíacos adversos maiores (ECAM) após ICP durante um acompanhamento de 5 anos em comparação aos homens.^15^ Dados obtidos do Global Burden Disease 2019 no Brasil, revelam que as DCV são a principal causa de morte tanto em mulheres quanto em homens; apesar da redução da mortalidade entre 1990 e 2019, a mortalidade proporcional é maior em mulheres.^2,16^

O objetivo do estudo foi avaliar mulheres com DAC obstrutiva (lesão > 50%), detectada por angiografia, admitidas por SCA ou DAC estável, submetidas a ICP contemporânea em um centro cardiovascular público no Brasil usando SLD e métodos de imagem/fisiologia quando indicado.

## Métodos

Este estudo foi conduzido de acordo com os princípios éticos descritos na Declaração de Helsinque, no Código de Nuremberg e nas Normas de Pesquisa Envolvendo Seres Humanos (Res. CNS 196/96) do Conselho Nacional de Saúde. O projeto de pesquisa foi submetido à revisão pelo Comitê de Ética em Pesquisa e recebeu aprovação tanto do hospital quanto dos pacientes ou seus responsáveis. O consentimento informado foi obtido de todos os pacientes.

### Desenho do estudo e pacientes

Trata-se de um estudo observacional, longitudinal, de coorte, com seguimento clínico prospectivo (FU). O estudo envolveu mulheres diagnosticadas com DAC estável e instável, caracterizadas por uma ou mais estenoses significativas. Essas mulheres foram submetidas a ICP com SLD entre janeiro de 2019 e dezembro de 2020. Todas as pacientes do sexo feminino, com mais de 18 anos, admitidas no departamento de cardiologia invasiva e submetidas a ICP com uma ou mais lesões > 50% no enxerto de artéria coronária nativa ou veia safena foram consecutivamente incluídas.

### Coleta de dados

Dados clínicos, demográficos e angiográficos dos pacientes foram coletados por meio de uma revisão abrangente de prontuários médicos físicos e eletrônicos armazenados no banco de dados da instituição. Essas informações foram registradas em um protocolo de pesquisa padronizado na forma de um questionário fechado. O FU clínico após ICP foi conduzida pelos pesquisadores envolvidos neste estudo. O FU incluiu uma revisão completa dos prontuários médicos, entrevistas por telefone e visitas no local sempre que possível. Todos os prontuários médicos disponíveis foram revisados para obter dados adicionais sobre fatores de risco emergentes e tradicionais, apresentação clínica, dados demográficos e angiográficos, bem como FU clínico e resultados cardiovasculares após intervenção.

### Pontos finais

Avaliar mulheres submetidas à ICP contemporânea na presença de SCA e DCI estável, analisando:

1. Resultados hospitalares: eventos cardíacos e cerebrovasculares adversos maiores (ECCAM) (morte por todas as causas, morte cardíaca, IM não fatal, acidente vascular cerebral e revascularização de emergência) e complicações hemorrágicas/vasculares.
2. ECCAM de médio prazo após alta hospitalar.
3. Identificar preditores para eventos hospitalares, ECCAM em médio prazo e sobrevida utilizando método de Kaplan Meier.

### Análise estatística

Variáveis contínuas foram descritas por médias e desvios-padrão, e variáveis categóricas por frequências absolutas e relativas. Modelos de regressão logística univariada avaliaram o efeito das variáveis sobre ECAM, apresentando odds ratios (ORs) com intervalos de confiança (ICs) de 95% e valores de p. Um modelo de regressão logística múltipla foi então ajustado para variáveis significativas da análise univariada. O nível de significância adotado na análise estatística foi de 5%.

Para o acompanhamento, a data inicial foi a da intervenção, e a data final foi a última ocorrência de acompanhamento ou evento. A análise de sobrevivência foi conduzida com ECAM como desfecho, apresentando razões de risco (HRs) com ICs de 95% e valores de p. Foram realizadas análises univariadas e multivariadas.

Todas as análises foram conduzidas utilizando o software R, versão 4.1.2.

## Resultados

Mil cento e quarenta e seis pacientes do sexo feminino (idade média de 65 anos) foram consecutivamente encaminhadas para ICP recomendada pelas diretrizes com SLD contemporâneo em artéria coronária nativa ou enxertos coronários devido à DAC estável ou instável.

Os pacientes exibiram uma alta prevalência de fatores de risco tradicionais, notavelmente hipertensão, dislipidemia, diabetes e histórico de tabagismo. Cerca de 30% das pacientes apresentavam história de IAM prévio e quase 70% foram internadas devido a SCA (Tabela 1).

**Tabela 1:** – Características basais (Demografia, Fatores de risco e Apresentação clínica)

	N = 1.146
**Idade, média (±DP)**	64,6 (±10)
**Raça**	
Branca	704 (61,4%)
Afrodescendente	422 (36,8%)
Outra	20 (1,8%)
**Hipertensão**	1.012 (88%)
**Diabetes**	545 (47,5%)
Medicação oral	333 (29%)
Insulina	212 (18%)
**Dislipidemia**	963 (85%)
**Obesidade**	178 (16%)
**Tabagismo**	
Atual	273 (24%)
Prévio	325 (28%)
**DRC**	
Não dialítico	151 (13%)
Dialítico	13 (1,1%)
**DAC familiar**	83 (7,2%)
**PAD**	78 (6,8%)
**AVE prévio**	67 (5,8%)
**IM prévio**	369 (32%)
**ICP prévia**	222 (19%)
**CRM prévia**	107 (9,3%)
**Apresentação clínica**	
DAC estável	338 (29,5%)
SCA	
IAMCSST	209 (18,2%)
IAMSSST	392 (34,2%)
AI	194 (17%)
Desconhecida	12 (1,1%)

DRC: doença renal crônica; DAC: doença arterial coronariana; AVC: acidente vascular cerebral; ICP: intervenção coronariana percutânea; CRM: cirurgia de revascularização miocárdica; DCI: doença cardíaca isquêmica; SCA: síndrome coronariana aguda; DAP: doença arterial periférica; IAM: infarto agudo do miocárdio; IAMCSST: infarto agudo do miocárdio com supra de ST; IAMSSST: infarto agudo do miocárdio sem supra de ST; AI: angina instável.

Fatores de risco emergentes e outras comorbidades não se mostraram prevalentes nesta população na avaliação restrospectiva. (Tabela 2).

**Tabela 2:** – Fatores de risco não tradicionais e outras comorbidades

	N= 1.146
Transtornos psiquiátricos	58 (5,1%)
Doenças reumatológicas / doenças autoimunes	44 (3,8%)
Neoplasias	36 (3,1%)
Doença hipertensiva gestacional	6 (0,5%)
Distúrbios endocrinológicos	142 (12,4%)
Doenças pulmonares	67 (5,9%)
Doenças hematológicas	11(1,0%)
Doenças infecciosas	9 (0,8%)
Distúrbios gastrointestinais	9 (0,8%)
Distúrbios endocrinológicos	142 (12,4%)
Doenças pulmonares	67 (5,9%)

A Tabela 3 ilustra as características relacionadas à ICP. Foram tratados 1.516 vasos em 1.146 pacientes, resultando em uma média de 1,3 vasos por paciente. A maioria dos casos envolveu o tratamento de um único vaso, enquanto 26% foram submetidos ao tratamento de dois vasos e 12,6% tiveram procedimentos estagiados. No total, foram implantados 1.725 stents, com média de 1,5 stents por paciente.

**Tabela 3:** – Características relacionadas ao ICP

Local de acesso	
Radial	636 (59%)
Femoral	435 (40%)
Braquial	10 (1%)
**Número de vasos tratados**	1.516
ADA	484 (32%)
ACD	405 (26,7%)
ACX	276 (18,2%)
TCE	55 (3,6%)
Outra	296 (19,5%)
**Número total de vasos tratados por paciente**	1,3
**Número de vasos tratados**	
1	764 (69%)
2	285 (26%)
3	49 (4,4%)
4	8 (0,7%)
**Stents implantados/paciente (SD)**	1,5 (±0,81)
**Número total de stents implantados**	1725
**Sucesso do procedimento por vaso tratado**	1492 (98,4%)
**Sucesso do procedimento por paciente**	1.081 (97,7%)
**Procedimentos estagiados**	145 (12,6%)
**Média de permanência hospitalar**	3,9 dias

ADA: artéria descendente anterior; ACD: artéria coronária direita; ACX: Artéria circunflexa; TCE: tronco da coronária esquerda.

A função ventricular esquerda, avaliada por ventriculografia durante angiografia ou por ecocardiografia transtorácica, indicou que a maioria dos pacientes apresentava função ventricular preservada ou disfunção leve (fração de ejeção > 40%). Disfunção ventricular moderada ou grave foi observada em 18% dos casos. A ICP foi predominantemente realizada por acesso radial, em quase 60% dos pacientes. A artéria descendente anterior foi o vaso mais tratado, seguida pelas artérias coronária direita e circunflexa. A ICP do tronco da coronária esquerda foi tratada em 3,6% dos pacientes.

A ICP demonstrou uma alta taxa de sucesso, com 97,7% dos pacientes e 98,4% dos vasos (Figura Central). As complicações foram classificadas em relacionadas ao procedimento, local de acesso e eventos clínicos. Cirurgia de emergência foi necessária em apenas três casos, que incluíram tamponamento cardíaco após ICP em dois pacientes e defeito do septo ventricular pós-infarto agudo do miocárdio (um caso).

Complicações clínicas ocorreram em 7,5% dos casos, incluindo infarto do miocárdio periprocedimento (IAM tipo 4A), acidente vascular cerebral e disfunção renal (piora aguda ou exacerbação da função renal na doença renal crônica - DRC). Todos os eventos cerebrovasculares foram reversíveis em 24 horas e classificados como ataques isquêmicos transitórios (AIT). Arritmias notáveis observadas incluíram fibrilação atrial e bloqueio atrioventricular completo, com um caso necessitando de implante de marcapasso. Além disso, dois pacientes desenvolveram COVID-19 durante a internação hospitalar, e seus casos não resultaram em complicações maiores. Em relação ao local de acesso, as complicações foram pouco frequentes: pseudoaneurisma da artéria femoral ocorreu em 1,0% dos casos, seguido por hematoma retroperitoneal e síndrome compartimental, todos tratados com sucesso (Tabela 4).

**Tabela 4:** – Resultados intra-hospitalares

	N = 1.146
**Complicações gerais**	163 (14,2%)
**Durante ICP**	63 (5,4%)
Perfuração Coronária	15 (1,3%)
Embolização	3 (0,3%)
*Slow flow/ no reflow*	5 (0,4%)
Trombose aguda	1(0,09%)
**Cirurgia de emergência**	3 (0,3%)
Defeito do septo ventricular (não relacionado à ICP)	1(0,09%)
Tamponamento cardíaco	2(0,18%)
**Relacionado ao acesso**	16 (1,3%)
Pseudoaneurisma femoral	11 (1,0%)
Sangramento	3 (0,3%)
Hematoma retroperitoneal	1 (0,09%)
Hemotransfusão	1 (0,09%)
Síndrome compartimental	1 (0,09%)
Trombose da artéria radial	1 (0,09%)
**Complicações clínicas**	84 (7,5%)
IAM periprocedimento	41 (3,6%)
AVC	5 (0,4%)
Disfunção renal	21 (1,8%)
Choque Cardiogênico	11 (1,2%)
Arritmias	8 (0,7%)
Sangramento gastrointestinal	1 (0,09%)
Infecções	15 (1,3%)
**Morte hospitalar**	14 (1,2%)

ICP: intervenção coronariana percutânea; IAM: infarto agudo do miocárdio; AVC: acidente vascular cerebral.

Análises univariadas e multivariadas foram conduzidas para avaliar a ocorrência de complicações hospitalares e eventos cardíacos e cerebrovasculares importantes (morte, acidente vascular cerebral, IAM e revascularização de emergência). Os preditores de complicações hospitalares na análise univariada incluíram idade, IAM prévio, hipertensão, presença de DRC e doença reumatológica, bem como o número de vasos tratados (> 2 vasos) e pelo menos uma falha de procedimento em um dos vasos tratados. O acesso radial foi identificado como um fator de proteção, embora não tenha mantido significância na análise ajustada.

Após análise multivariada ajustada, apenas idade, DRC e pelo menos uma falha de procedimento em um dos vasos tratados permaneceram preditores do risco de complicações hospitalares (Apêndice – Tabela 5).

Na análise multivariada, identificamos vários fatores preditivos para a ocorrência de ECCAM intra-hospitalar. Um histórico de acidente vascular cerebral, DRC e pelo menos uma falha de procedimento durante intervenção percutânea emergiram como contribuintes significativos (Apêndice – Tabela 6).

Durante a hospitalização e após a alta, os pacientes receberam tratamento seguindo as terapias recomendadas pelas diretrizes, incluindo Terapia Antiplaquetária Dupla por 6-12 meses, estatinas, betabloqueadores, inibidores da ECA/BRAs e medicamentos antidiabéticos disponíveis no sistema de saúde pública. Além disso, modificações no estilo de vida, cessação do tabagismo, dieta saudável e exercícios foram fortemente recomendados.

Para o FU, reunimos informações abrangentes e consistentes para 1047 participantes, incluindo a data da alta, as datas dos eventos mencionados e a data do último acompanhamento. Foram excluídos, 14 mortes hospitalares e a perda de seguimento de 85 pacientes. A análise de sobrevivência foi conduzida com esta coorte. Durante o período de FU, a mortalidade por todas as causas ocorreu em 5,3%, com 3,5% atribuídos a causas cardíacas. SCA recorrente foi observada em quase 8% dos pacientes, e procedimentos adicionais de revascularização (relacionados a SCA, reestenose e progressão de DAC) foram identificados em 5,5% dos casos.

**Figura 1:**
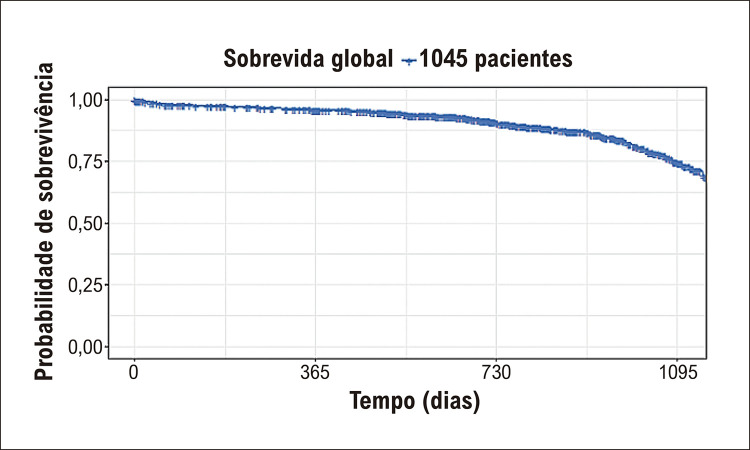
– Curva de Kaplan Meier.

O tempo médio de FU foi de 576,2 dias (DP: ±423 dias). O tempo médio de sobrevida dos participantes foi de 1249 dias, como demonstrado pela curva de Kaplan-Meier na Figura 1. Ao final o período de seguimento, 86% dos pacientes permaneceram livres de ECCAM.

Os fatores preditivos para eventos cardíacos e cerebrovasculares graves identificados durante o período de FU foram admissão hospitalar por SCA para a ICP índice e a ocorrência de eventos cardíacos e cerebrovasculares graves durante a hospitalização (ver Apêndice – Tabela 7).

## Discussão

Este estudo pioneiro realizado no Brasil analisou ICP com implante de stent farmacológico em mais de 1.000 mulheres com DAC estável e SCA em um hospital público terciário. O objetivo primário foi avaliar os principais desfechos cardiovasculares intra-hospitalares e de médio prazo, juntamente com a identificação de seus preditores de risco.

Vale ressaltar que este estudo começou em janeiro de 2019 e foi, posteriormente, realizado durante o período desafiador da pandemia de COVID-19 (2020 a 2022). Durante esse período, os serviços públicos de saúde em todo o mundo enfrentaram desafios significativos, sendo sobrecarregados pelo gerenciamento simultâneo de infecções por coronavírus e suas graves consequências clínicas, além de abordar doenças cardiovasculares e outras enfermidades.

Os principais achados do nosso estudo indicam que a ICP com SLD, juntamente com o tratamento clínico otimizado para condições estáveis e instáveis em mulheres, produz excelentes resultados hospitalares e demonstra resultados clínicos encorajadores em médio prazo. Os fatores que preveem eventos cardiovasculares importantes precoces incluíram acidente vascular cerebral anterior, a presença de DRC e falha da ICP. Além disso, a admissão por SCA antes da ICP índice e ECCAM hospitalar foram identificados como preditores para resultados em médio prazo, alinhando-se com os resultados relatados em estudos anteriores.

Em termos de características clínicas, a média de idade foi de 64,6 anos, com notável prevalência de fatores de risco tradicionais e comorbidades associadas. Recentemente, se tem chamado a atenção para a presença de fatores de risco emergentes específicos para mulheres, relacionados ao sexo e gênero, que contribuem para a fisiopatologia da DCI neste subgrupo.^4,17^ Em nosso estudo, a prevalência desses fatores pareceu baixa e pode ter sido subestimada, o que pode estar ligado à natureza retrospectiva da coleta de dados e ao viés de seleção. Os dados foram coletados retrospectivamente, e há uma possibilidade de viés de seleção, pois apenas pacientes com lesões coronárias > 50% submetidos a ICP foram incluídos. Esse critério de exclusão pode ter negligenciado certas condições clínicas, como MINOCA, INOCA e ANOCA, que envolvem diversas fisiopatologias que compreendem vários fatores biológicos e psicossociais.

No entanto, conseguimos identificar fatores de risco não tradicionais e outras doenças não cardiovasculares nesta população (Tabela 2).

Na maioria dos casos, a ICP foi realizada na presença de SCA, preferencialmente por via radial. Estudos indicam que, em comparação aos homens, as mulheres enfrentam um risco maior de complicações vasculares e sangramento periprocedimento, particularmente em condições agudas. Consequentemente, as diretrizes atuais e estudos randomizados recomendam a abordagem radial sempre que tecnicamente viável, visando atenuar complicações vasculares, sangramento e eventos isquêmicos.^18^

Em relação às características angiográficas, apesar da alta prevalência de fatores de risco para DAC, 69% dos pacientes apresentaram doença uniarterial, sendo a artéria descendente anterior a mais frequentemente tratada. A ICP do tronco da coronária esquerda desprotegido (3,6%) e doença de triarterial (5%) foi pouco frequente, sugerindo que os pacientes com doenças mais complexas foram provavelmente encaminhados para cirurgia de revascularização miocárdica. A taxa de sucesso da ICP (por paciente e vaso tratado) foi notavelmente alta, 98%, possivelmente atribuída à presença de doença coronária menos complexa, com uma média de 1,5 stents implantados por paciente e poucos procedimentos estagiados.

As mulheres são reconhecidamente portadoras de vasos menores e maior tortuosidade coronária, aumentando sua suscetibilidade a complicações durante a ICP, como dissecções e perfurações,^7^ que ocorreram em menos de 5% dos nossos pacientes e foram tratados com sucesso. Devido a essas características anatômicas, uma avaliação cuidadosa do diâmetro do vaso e da extensão da lesão é crucial, normalmente conduzida por angiografia e por operadores experientes. Em casos mais desafiadores, métodos de fisiologia coronária e imagens intravasculares foram empregados (aproximadamente 10% dos casos) para orientar decisões terapêuticas e implantação de stents, garantindo procedimentos tecnicamente ideais com complicações mínimas.

Em nossa amostra de pacientes, complicações clínicas periprocedimento foram raras e efetivamente gerenciadas: pseudoaneurisma femoral ocorreu em 1%, enquanto sangramento grave foi observado em apenas 0,3%. IAM periprocedimento ocorreu em 3,6% dos casos, AIT em 0,4% e lesão renal aguda pós-contraste em 1,8%. A mortalidade hospitalar permaneceu baixa em 1,2%, mesmo na presença de SCA e alta prevalência de comorbidades associadas.

Esses resultados ressaltam a proficiência técnica da equipe do hospital terciário de cardiologia, que lida com um alto volume de procedimentos. Eles demonstram uma preferência pelo acesso radial, a adoção de estratégias de nefroproteção empregando contrastes de baixa/isoosmolaridade em pequenos volumes, hidratação periprocedimento sistemática para pacientes com depuração < 60 ml/kg/min e a utilização universal de stents farmacológicos de nova geração.

Esses achados divergem dos apresentados por outros pesquisadores, que indicam taxas de mortalidade elevadas, particularmente em SCA. Uma metanálise abrangendo estudos pós-SCA revela uma taxa de mortalidade aproximada de 5%.^19^ Os piores resultados mais pobres podem estar ligados a disparidades no acesso a serviços de saúde, diagnóstico tardio e tratamento inadequado. Estudos também indicam que as mulheres são submetidas a menos procedimentos invasivos (como ICP e cirurgia) e recebem menos medicamentos recomendados pelas diretrizes para o tratamento de doença cardíaca isquêmica.^20^

Em 2015, Bavishi et al. conduziram uma metanálise compreendendo 48 estudos envolvendo 103.895 pacientes submetidos a ICP após infarto do miocárdio com elevação do segmento ST. Sua análise das diferenças de gênero na mortalidade revelou que as mulheres apresentaram uma taxa de mortalidade mais alta, particularmente no curto prazo, enfatizando o papel crucial da terapia rápida na mitigação de resultados adversos.^21^ Isso ressalta a necessidade de implementar protocolos padronizados de departamento de emergência e fornecer educação médica contínua para lidar com as disparidades substanciais na abordagem de pacientes do sexo masculino e feminino, especialmente em cenários instáveis.

Os preditores de complicações hospitalares em nosso estudo se alinham com aqueles documentados na literatura. Idade avançada, presença de DRC e ICP malsucedida são comumente associados a um prognóstico pior (Tabela 6).

A idade avançada está associada ao aumento de comorbidades, risco elevado de complicações clínicas e anatomia coronária mais complexa. DRC e ICP malsucedida foram identificadas como preditores de ECCAM hospitalar, contribuindo para resultados clínicos adversos iniciais com risco elevado de diálise, descompensação cardíaca, recorrência de angina e internações hospitalares prolongadas. Mazurek et al. demonstraram que a falha da ICP é um fator de risco independente para aumento da mortalidade em SCA.^22^ Barbash et al. apoiaram esta descoberta, revelando adicionalmente que o gênero feminino prevê independentemente a falha do procedimento no infarto do miocárdio com supradesnivelamento do segmento ST (OR 1,54; IC 95% [1,01-2,38]).^23^ Os preditores de ECCAM durante o acompanhamento de 2 anos foram admissão por SCA e a presença de eventos hospitalares.

No acompanhamento durante o seguimento médio de 576 dias, 15% dos nossos pacientes apresentaram desfechos cardíacos e cerebrovasculares importantes, incluindo morte cardíaca em 3,5% e SCA recorrente em 8%. A taxa de sobrevida livre de eventos foi de 86%. Esses desfechos significativos se assemelham muito aos relatados por Mehran et al. após um acompanhamento de 2 anos no estudo Spirit Women, que randomizou mulheres para receber SLD com sirolimus ou everolimus.^24^

### Limitações

A principal limitação do nosso estudo está na falta de comparação com homens tratados por ICP durante o mesmo período, o que seria altamente desejável para verificar se há de fato diferenças nos resultados clínicos imediatos e tardios após ICP entre eles.

## Conclusão

Neste estudo contemporâneo envolvendo 1146 pacientes do sexo feminino tratadas por ICP de acordo com terapias recomendadas por diretrizes e acompanhadas por aproximadamente 2 anos, alcançamos resultados hospitalares e de médio prazo altamente promissores. Vale ressaltar que esta pesquisa foi conduzida em um hospital público terciário, em parte durante a pandemia de COVID-19, destacando os desafios inerentes a este contexto.

## References

[1] Aggarwal NR, Patel HN, Mehta LS, Sanghani RM, Lundberg GP, Lewis SJ (2018). Sex Differences in Ischemic Heart Disease: Advances, Obstacles, and Next Steps. Circ Cardiovasc Qual Outcomes.

[2] Vogel B, Acevedo M, Appelman Y, Merz CNB, Chieffo A, Figtree GA (2021). The Lancet Women and Cardiovascular Disease Commission: Reducing the Global Burden by 2030. Lancet.

[3] Mehilli J, Presbitero P (2020). Coronary Artery Disease and Acute Coronary Syndrome in Women. Heart.

[4] Geraghty L, Figtree GA, Schutte AE, Patel S, Woodward M, Arnott C (2021). Cardiovascular Disease in Women: From Pathophysiology to Novel and Emerging Risk Factors. Heart Lung Circ.

[5] Minissian MB, Mehta PK, Hayes SN, Park K, Wei J, Merz CNB (2022). Ischemic Heart Disease in Young Women: JACC Review Topic of the Week. J Am Coll Cardiol.

[6] Merz CNB, Shaw LJ, Reis SE, Bittner V, Kelsey SF, Olson M (2006). Insights from the NHLBI-Sponsored Women's Ischemia Syndrome Evaluation (WISE) Study: Part II: Gender Differences in Presentation, Diagnosis, and outcome with regard to Gender-Based Pathophysiology of Atherosclerosis and Macrovascular and Microvascular Coronary Disease. J Am Coll Cardiol.

[7] Yahagi K, Davis HR, Arbustini E, Virmani R (2015). Sex Differences in Coronary Artery Disease: Pathological Observations. Atherosclerosis.

[8] Gaudino M, Di Franco A, Cao D, Giustino G, Merz CNB, Fremes SE (2022). Sex-Related Outcomes of Medical, Percutaneous, and Surgical Interventions for Coronary Artery Disease: JACC Focus Seminar 3/7. J Am Coll Cardiol.

[9] Wenger NK (2024). The Feminine Face of Heart Disease 2024. Circulation.

[10] Iribarren A, Diniz MA, Merz CNB, Shufelt C, Wei J (2022). Are We Any WISER Yet? Progress and Contemporary Need for Smart Trials to Include Women in Coronary Artery Disease Trials. Contemp Clin Trials.

[11] Roumeliotis A, Claessen BE, Sartori S, Cao D, Qiu H, Camaj A (2021). Impact of Sex on Long-Term Cardiovascular Outcomes of Patients Undergoing Percutaneous Coronary Intervention for Acute Coronary Syndromes. Catheter Cardiovasc Interv.

[12] Batchelor W, Kandzari DE, Davis S, Tami L, Wang JC, Othman I (2017). Outcomes in Women and Minorities Compared with White Men 1 Year after Everolimus-Eluting Stent Implantation: Insights and Results from the PLATINUM Diversity and PROMUS Element Plus Post-Approval Study Pooled Analysis. JAMA Cardiol.

[13] Lichtman JH, Leifheit EC, Safdar B, Bao H, Krumholz HM, Lorenze NP (2018). Sex Differences in the Presentation and Perception of Symptoms Among Young Patients with Myocardial Infarction: Evidence from the VIRGO Study (Variation in Recovery: Role of Gender on Outcomes of Young AMI Patients). Circulation.

[14] Diercks DB, Owen KP, Kontos MC, Blomkalns A, Chen AY, Miller C (2010). Gender Differences in Time to Presentation for Myocardial Infarction Before and after a National Women's Cardiovascular Awareness Campaign: A Temporal Analysis from the Can Rapid Risk Stratification of Unstable Angina Patients Suppress ADverse Outcomes with Early Implementation (CRUSADE) and the National Cardiovascular Data Registry Acute Coronary Treatment and Intervention Outcomes Network-Get with the Guidelines (NCDR ACTION Registry-GWTG). Am Heart J.

[15] Anderson ML, Peterson ED, Brennan JM, Rao SV, Dai D, Anstrom KJ (2012). Short- and Long-Term Outcomes of Coronary Stenting in Women versus Men: Results from the National Cardiovascular Data Registry Centers for Medicare & Medicaid Services Cohort. Circulation.

[16] Oliveira GMM, Brant LCC, Polanczyk CA, Malta DC, Biolo A, Nascimento BR (2022). Cardiovascular Statistics - Brazil 2021. Arq Bras Cardiol.

[17] Agarwala A, Michos ED, Samad Z, Ballantyne CM, Virani SS (2020). The Use of Sex-Specific Factors in the Assessment of Women's Cardiovascular Risk. Circulation.

[18] Gargiulo G, Ariotti S, Vranckx P, Leonardi S, Frigoli E, Ciociano N (2018). Impact of Sex on Comparative Outcomes of Radial versus Femoral Access in Patients with Acute Coronary Syndromes Undergoing Invasive Management: Data from the Randomized MATRIX-Access Trial. JACC Cardiovasc Interv.

[19] Guo Y, Yin F, Fan C, Wang Z (2018). Gender Difference in Clinical Outcomes of the Patients with Coronary Artery Disease after Percutaneous Coronary Intervention: A Systematic Review and Meta-Analysis. Medicine (Baltimore).

[20] Redfors B, Angerås O, Råmunddal T, Petursson P, Haraldsson I, Dworeck C (2015). Trends in Gender Differences in Cardiac Care and Outcome after Acute Myocardial Infarction in Western Sweden: A Report from the Swedish Web System for Enhancement of Evidence-Based Care in Heart Disease Evaluated According to Recommended Therapies (SWEDEHEART). J Am Heart Assoc.

[21] Bavishi C, Bangalore S, Patel D, Chatterjee S, Trivedi V, Tamis-Holland JE (2015). Short and Long-Term Mortality in Women and Men Undergoing Primary Angioplasty: A Comprehensive Meta-Analysis. Int J Cardiol.

[22] Mazurek M, Kowalczyk J, Lenarczyk R, Swiatkowski A, Kowalski O, Sedkowska A (2011). The Impact of Unsuccessful Percutaneous Coronary Intervention on Short- and Long-Term Prognosis in STEMI and NSTEMI. Catheter Cardiovasc Interv.

[23] Barbash IM, Ben-Dor I, Torguson R, Maluenda G, Xue Z, Gaglia MA (2012). Clinical Predictors for Failure Of Percutaneous Coronary Intervention in ST-Elevation Myocardial Infarction. J Interv Cardiol.

[24] Baber U, Kini AS, Gukathasan N, Mehran R (2012). Abstract 18379: Impact of Gender on Adverse Cardiovascular Events Following Percutaneous Coronary Intervention: Pooled Analysis from the SPIRIT II, III and IV trials. Circulation.

