# Supplementary material for: Resultados da Intervenção Coronariana Percutânea Recomendada pelas Diretrizes em Mulheres com Doença Arterial Coronariana Obstrutiva: um Estudo de Coorte Longitudinal
Source: Arq Bras Cardiol. 2025 Jan 22;122(1):e20240249. [Article in Portuguese] doi: 10.36660/abc.20240249 (PMC11805569; doi:10.36660/abc.20240249)
Supplement: Translation

# Translation: Outcomes of Guideline-Recommended Percutaneous Coronary Intervention in Women with Obstructive Coronary Artery Disease: A Longitudinal Cohort Study

**DOI:** 10.36660/abc.20240249i

**Authors:** Tacianne Rolemberg Braga Delamain, José Henrique Herrmann Delamain, Sergio Luiz Navarro Braga, Ricardo Costa, Dimytri Alexandre Alvim de Siqueira, Fausto Feres, Marinella Patrizia Centemero

**Affiliations:** 1 Brazil Instituto Dante Pazzanese de Cardiologia São Paulo, SP – Brazil

**Keywords:** Percutaneous Coronary Intervention, Women, Coronary Artery Disease, Risk Factors

## Abstract

**Background:**

Previous studies have shown that women with coronary artery disease (CAD) are less likely to undergo angiography and have less favorable outcomes after percutaneous coronary intervention (PCI).

**Objectives:**

Assess the outcomes of women with acute coronary syndrome (ACS) and stable CAD (lesion>50%) treated with contemporary PCI using DES.

**Methods:**

Observational, longitudinal cohort study with prospective follow-up included all female patients ≥ 18 years admitted at a tertiary public cardiovascular center in Brazil from January 2019 to December 2020. The level of significance adopted in the statistical analysis was 5%.

**Results:**

1146 women (average age 65 years) underwent guideline-recommended PCI. Risk factors were frequent (hypertension: 88%, dyslipidemia: 85%, diabetes: 47.5%), and 69% were admitted due to ACS. Radial access was used in 59% of patients; 1516 vessels were treated with 1725 stents implanted (1.5 stents/patient). PCI was successful in 97.7%, in-hospital death occurred in 1.2%, peri-procedural MI in 3.6%, and TIA in 0.4%. Predictors of in-hospital major adverse cardiac and cerebrovascular events (MACCE): previous stroke (OR: 2.97; CI: 1.06-7.15; p= 0.023), CKD (OR: 3.11; CI: 1.49-6.20; p= 0.002), and at least one procedural failure during PCI (OR: 10.2; CI: 1.17-5.9; p<0.001). The average follow-up was 576.2 days in 1047 patients. All-cause mortality occurred in 5.3%, cardiac death in 3.5%, recurrent ACS in 8%, and additional revascularization procedures in 5.5%. The predictors for MACCE during FU were hospital admission for ACS for the index PCI (OR: 1.58; HR: 1.06-2.35; p=0.023) and the presence of MACCE during hospitalization (OR: 6.66; HR: 2.42- 18.3; p< 0.001).

**Conclusion:**

In this pioneering study involving 1146 patients treated by contemporary PCI and followed for almost 2 years, we obtained very encouraging in-hospital and mid-term results.

## Introduction

Cardiovascular diseases are a significant threat to women, with coronary heart disease (CHD) being the main factor responsible for this concerning trend.^1^ While the impact of CHD on men has been extensively documented, recent data underscore its substantial risk to women’s health.^2,3^ On a closer look, it becomes evident that both biological sex and sociocultural gender factors play a significant role in the development of CHD and in shaping its path in women. The intricate interaction between sex and gender has been increasingly recognized due to its profound implications.^4,5^ Biological factors, including smaller coronary arteries and a higher incidence of non-obstructive ischemic heart disease (IHD), undoubtedly contribute to the unique presentation of CHD and, therefore, to the outcomes of its treatment in women.^6,7^ Additionally, the gender and sociocultural aspect has a subtle yet major influence. Factors such as social stressors, barriers to accessing health services, and atypical symptoms disproportionately affect women and have been increasingly recognized for their role in delaying diagnosis and subsequent treatment.^8,9^ In addition, the historical predominance of male patients in clinical trials has influenced the development of treatment guidelines, resulting in the under-treatment of IHD in women.^10^

Specifically concerning percutaneous coronary intervention (PCI) in women, worse outcomes have been reported, even in the contemporary era using drug-eluting stents (DES).^11,12^ Evidence suggests that women receive guideline-recommended diagnostics and therapies less frequently than men, potentially leading to a significant impact on outcomes after PCI.^13,14^ Recent research has shown that women have a higher incidence of major adverse cardiac events (MACE) post PCI during a 5-year follow-up compared to men.^15^ Data obtained from Global Burden Disease 2019 in Brazil reveal that CVD is the leading cause of death in both women and men; besides decreasing by 50.6% between 1990 and 2019, proportional mortality was higher in women.^2,16^

The purpose of the study was to evaluate women presenting with acute coronary syndrome (ACS) and stable IHD with obstructive CAD (> 50%) detected on an angiogram and treated with contemporary PCI in a public cardiovascular center in Brazil using DES and imaging/physiology methods when indicated.

## Methods

This study was conducted in accordance with the ethical principles outlined in the Declaration of Helsinki, the Nuremberg Code, and the Norms of Research Involving Human Beings (Res. CNS 196/96) of the National Health Council. The research project underwent review by the Research Ethics Committee and received approval from both the hospital and the patients or their guardians. Informed consent was obtained from all patients.

### Study design and patients

This is an observational, longitudinal cohort study with prospective clinical follow-up (FU). The study involved women diagnosed with stable and unstable IHD, characterized by one or more significant stenoses. These women underwent PCI with DES between January 2019 and December 2020. All female patients aged over 18, admitted to the invasive cardiology department, and undergoing PCI with one or more lesions > 50% in the native coronary artery or saphenous vein graft were consecutively included. The inclusion criteria focused on lesions suitable for coronary stent implantation and clinically driven cases.

## Data collection

Clinical, demographic, and angiographic data of the patients were collected through a comprehensive review of both physical and electronic medical records stored in the institution’s database. This information was recorded in a standardized research protocol in the form of a closed questionnaire. Clinical FU after PCI was conducted by the researchers involved in this study. The FU included a thorough review of medical records, telephone interviews, and on-site visits whenever possible. All available medical records were reviewed to obtain additional data on emerging and traditional risk factors, clinical presentation, demographic, and angiographic data, as well as clinical FU and cardiovascular outcomes after intervention.

### Endpoints

To evaluate women undergoing contemporary PCI in the presence of ACS and stable IHD, analyzing:

1. Hospital outcomes: major adverse cardiac and cerebrovascular events (MACCE) (all-cause death, cardiac death, non-fatal MI, stroke, and emergency revascularization) and hemorrhagic/vascular complications.
2. Mid-term MACCE after hospital discharge.
3. Identify predictors for in-hospital events, mid-term MACCE, and Kaplan-Meier survival.

### Statistical analysis

Continuous variables were described using means and standard deviations, and categorical variables using absolute and relative frequencies. Univariate logistic regression models assessed the effect of variables on MACE, presenting odds ratios (ORs) with 95% confidence intervals (CIs) and p-values. A multiple logistic regression model was then adjusted for significant variables from the univariate analysis. The level of significance adopted in the statistical analysis was 5%.

For follow-up, the initial date was the intervention date, and the final date was the last follow-up or event occurrence. Survival analysis was conducted with MACE as the outcome, presenting hazard ratios (HRs) with 95% CIs and p-values. Both univariate and multivariate analyses were performed.

All analyses were conducted using R software, version 4.1.2.

## Results

One thousand one hundred forty-six female patients (average age 65 years) were consecutively referred for guideline-recommended PCI with contemporary DES in native coronary artery or coronary grafts due to stable or unstable IHD.

The patients exhibited a high prevalence of traditional risk factors, notably hypertension, dyslipidemia, diabetes, and a history of smoking. One-third had a prior MI, and nearly 70% were admitted due to ACS (Table 1).

**Table 1:** – Baseline characteristics (Demographics, Risk factors, and Clinical presentation)

	N = 1,146
**Age, mean (±SD)**	64.6 (±10)
**Race**	
white	704 (61.4%)
afro-descendent	422 (36.8%)
other	20 (1.8%)
**Hypertension**	1.012 (88%)
**Diabetes**	545 (47.5%)
Oral medication	333 (29%)
Insulin	212 (18%)
**Dyslipidemia**	963 (85%)
**Obesity**	178 (16%)
**Smoking**	
current	273 (24%)
former	325 (28%)
**CKD**	
non-dialytic	151 (13%)
dialytic	13 (1.1%)
**FAMILY CAD**	83 (7.2%)
**PAD**	78 (6.8%)
**Prior stroke**	67 (5.8%)
**Prior MI**	369 (32%)
**Prior PCI**	222 (19%)
**Previous CABG**	107 (9.3%)
**Clinical presentation**	
Stable IHD	338 (29.5%)
ACS	
STEMI	209 (18.2%)
Non-STEMI	392 (34.2%)
UA	194 (17%)
Unknown	12 (1.1%)

CKD: chronic kidney disease; CAD: coronary artery disease; PAD: peripheral artery disease; MI: Myocardial infarction; PCI: percutaneous coronary intervention; CABG: coronary artery bypass grafting; IHD: ischemic heart disease; ACS: acute coronary syndrome; STEMI: ST-elevation myocardial infarction; Non-STEMI: non-ST-elevation myocardial infarction; UA: Unstable angina.

Emerging risk factors and other comorbidities were not as prevalent in this population (Table 2).

**Table 2:** – Non-traditional risk factors and other comorbidities

	N= 1,146
Psychiatric disorders	58 (5.1%)
Rheumatologic diseases / autoimmune diseases	44 (3.8%)
Neoplasias	36 (3.1%)
Gestational hypertensive disease	6 (0.5%)
Endocrinological disorders	142 (12.4%)
Lung diseases	67 (5.9%)
Blood diseases	11(1.0%)
Infectious diseases	9 (0.8%)
Gastrointestinal disorders	9 (0.8%)
Endocrinological disorders	142 (12.4%)
Lung diseases	67 (5.9%)

Table 3 illustrates the characteristics related to percutaneous coronary intervention (PCI). A total of 1.516 vessels were treated in 1.146 patients, resulting in an average of 1.3 vessels per patient. The majority of cases involved the treatment of a single vessel, while 26% underwent treatment for two vessels, and 12.6% had staged procedures. In total, 1.725 stents were implanted, averaging 1.5 stents per patient.

**Table 3:** – PCI-related characteristics

Access site	
Radial	636 (59%)
Femoral	435 (40%)
Brachial	10 (1%)
**Number of treated vessels**	1.516
LAD	484 (32%)
RCA	405 (26.7%)
LCX	276 (18.2%)
Left main	55 (3.6%)
Other	296 (19.5%)
**Total number of treated vessels per patient**	1.3
**Number of treated vessels**	
1	764 (69%)
2	285 (26%)
3	49 (4.4%)
4	8 (0.7%)
**Stents implanted/patient (SD)**	1.5 (±0.81)
**Total number of implanted stents**	1725
**Procedure success per vessel treated**	1492 (98.4%)
**Procedural success per patient**	1.081 (97.7%)
**Staged procedures**	145 (12.6%)
**Average hospital stay**	3.9 days

LAD: left anterior descending; RCA: right coronary artery; LCX: left circumflex artery.

Left ventricular function, evaluated through ventriculography during diagnostic coronary angiography or transthoracic echocardiography, indicated that most patients exhibited preserved ventricular function or mild dysfunction (ejection fraction > 40%). Moderate or severe ventricular dysfunction was observed in 18% of cases. PCI was predominantly carried out via radial access, accounting for nearly 60% of patients. The anterior descending artery was the most treated vessel, followed by the right coronary and circumflex arteries. Unprotected left main PCI was treated in 3.6% of patients.

PCI demonstrated a high success rate, with 97.7% of patients and 98.4% of vessels (Central Illustration). Complications were classified into procedure-related, access site, and clinical events. Emergency surgery was required in only three cases, which included cardiac tamponade following PCI in two patients and ventricular septal defect post-acute myocardial infarction (one case).

Clinical complications occurred in 7.5% of cases, including peri-procedural myocardial infarction (Type 4A AMI), stroke, and renal dysfunction (acute worsening or exacerbation of renal function in chronic kidney disease – CKD). All cerebrovascular events were reversible within 24 hours and classified as transient ischemic attacks (TIA). Noteworthy arrhythmias observed included atrial fibrillation and complete atrioventricular block, with one case requiring pacemaker implantation. Additionally, two patients developed COVID-19 during their hospital stay, and their cases did not result in major complications. Concerning the access site, complications were infrequent: femoral artery pseudoaneurysm occurred in 1.0% of cases, followed by retroperitoneal hematoma and compartmental syndrome, all of which were successfully managed (Table 4).

**Table 4:** – In-hospital results

	N = 1,146
**Overall complications**	163 (14.2%)
**During PCI**	63 (5.4%)
Coronary Perforation	15 (1.3%)
Embolization	3 (0.3%)
Slow flow/No Reflow	5 (0.4%)
Acute thrombosis	1(0.09%)
**Emergency surgery**	3 (0.3%)
Ventricular septal defect (not PCI-related)	1(0.09%)
Cardiac tamponade	2(0.18%)
**Access related**	16 (1.3%)
Femoral Pseudoaneurysm	11 (1.0%)
Bleeding	3 (0.3%)
Retroperitoneal Hematoma	1 (0.09%)
Hemotransfusion	1 (0.09%)
Compartmental syndrome	1 (0.09%)
Radial artery thrombosis	1 (0.09%)
**Clinical complications**	84 (7.5%)
Peri-procedural MI	41 (3.6%)
Stroke	5 (0.4%)
Renal dysfunction	21 (1.8%)
Cardiogenic Shock	11 (1.2%)
Arrhythmias	8 (0.7%)
GI bleeding	1 (0.09%)
Infections	15 (1.3%)
**Hospital death**	14 (1.2%)

Univariate and multivariate analyses were conducted to evaluate the occurrence of in-hospital complications and major cardiac and cerebrovascular events (death, stroke, MI, and emergency revascularization). Predictors of in-hospital complications in the univariate analysis included age, prior MI, hypertension, the presence of CKD, and rheumatologic disease, as well as the number of treated vessels (>2 vessels) and at least one procedural failure in one of the treated vessels. Radial access was identified as a protective factor, although it did not maintain significance in the adjusted analysis.

After adjusted multivariate analysis, only age, CKD, and at least one procedural failure in one of the treated vessels remained predictors of the risk for in-hospital complications (Appendix – Table 5).

In the multivariate analysis, we identified several predictive factors for the occurrence of in-hospital MACCE. A history of stroke, CKD, and at least one procedural failure during percutaneous intervention emerged as significant contributors (Appendix – Table 6).

Throughout hospitalization and post-discharge, patients received treatment following guideline-recommended therapies, including Dual Antiplatelet Therapy for 6-12 months, statins, beta-blockers, ACE inhibitors/ARBs, and antidiabetic medications available in the public health system. Additionally, lifestyle modifications, smoking cessation, a healthy diet, and exercise were strongly recommended.

We gathered comprehensive and consistent information for 1047 participants, including the date of discharge, the dates of the mentioned events, and the date of the last follow-up. Fourteen in-hospital deaths and eighty-five participants lost to follow-up were excluded. Survival analysis was conducted with this cohort. During the FU period, all-cause mortality occurred in 5.3%, with 3.5% attributed to cardiac causes. Recurrent ACS was observed in nearly 8% of patients, and additional revascularization procedures (related to ACS, restenosis, and CAD progression) were identified in 5.5% of cases.

The average FU time was 576.2 days (SD: 423 days). The median survival time of the participants was 1249 days, and the Kaplan-Meier curve is depicted in Figure 1. At the conclusion of the follow-up period, 86% of patients remained free from MACCE.

**Figure 1:** – Kaplan Meier Curve.

The predictive factors for major cardiac and cerebrovascular events identified during the FU period were hospital admission for ACS during the index PCI procedure and the occurrence of major cardiac and cerebrovascular events during hospitalization (See Appendix – Table 7).

## Discussion

This pioneering study conducted in Brazil analyzed PCI with drug-eluting stent implantation in over 1,000 women with stable coronary artery disease and ACS at a tertiary public hospital. The primary objective was to evaluate major in-hospital and medium-term cardiovascular outcomes, along with identifying their risk predictors.

It is noteworthy that this study started in January 2019 and was subsequently carried out during the challenging period of the COVID-19 pandemic (2020 to 2022). During this time, public healthcare services worldwide faced significant challenges, being overwhelmed by the simultaneous management of coronavirus infections and their severe clinical consequences, in addition to addressing cardiovascular diseases and other illnesses.

The primary findings of our study indicate that PCI with DES, coupled with optimized clinical treatment for both stable and unstable conditions in women, yields excellent in-hospital outcomes and demonstrates encouraging mid-term clinical results. Factors predicting early major cardiovascular events included previous stroke, the presence of chronic kidney disease, and PCI failure. Additionally, admission for ACS before the index PCI and in-hospital MACCE were identified as predictors for mid-term outcomes, aligning with results reported in earlier studies.

In terms of clinical characteristics, the mean age was 64.6 years, with a notable prevalence of traditional risk factors and associated comorbidities. Recently, attention has been drawn to the presence of emerging risk factors specific to women, related to sex and gender, which contribute to the pathophysiology of IHD in this subgroup.^4,17^ In our study, the prevalence of these factors seemed low and might have been underestimated, which could be linked to the retrospective nature of data collection and selection bias. Data were gathered retrospectively, and there is a possibility of selection bias as only patients with coronary lesions > 50% undergoing PCI were included. This exclusion criterion might have overlooked certain clinical conditions such as MINOCA, INOCA, and ANOCA, which involve diverse pathophysiologies comprising various biological and psychosocial factors.

Nevertheless, we were able to identify non-traditional risk factors and other non-cardiovascular diseases in this population (Table 2).

In most cases, PCI was performed in the presence of ACS, preferably via radial access. Studies indicate that, in comparison to men, women face a higher risk of vascular complications and peri-procedural bleeding, particularly in acute conditions. Consequently, current guidelines and randomized studies recommend the radial approach whenever technically feasible, aiming to mitigate vascular complications, bleeding, and ischemic events.^18^

Regarding angiographic characteristics, despite the high prevalence of risk factors for CAD, 69% of patients exhibited single-vessel disease, with the anterior descending artery being the most frequently treated. PCI of unprotected left main (3.6%) and triple-vessel disease (5%) was infrequent, suggesting that patients with more complex diseases were likely referred for coronary artery bypass grafting. The success rate of PCI (per patient and vessel treated) was notably high at 98%, possibly attributed to the presence of less complex coronary disease, with an average of 1.5 stents implanted per patient and few staged procedures.

Women are recognized to have smaller vessels and greater coronary tortuosity, increasing their susceptibility to complications during PCI, such as dissections and perforations,^7^ which occurred in less than 5% of our patients and were successfully managed. Due to these anatomical characteristics, a careful assessment of vessel diameter and lesion extent is crucial, typically conducted through angiography and by experienced operators. In more challenging cases, coronary physiology methods and intravascular imaging have been employed (approximately 10% of cases) to guide therapeutic decisions and stent implantation, ensuring technically optimal procedures with minimal complications.

In our patient sample, peri-procedural clinical complications were rare and effectively managed: femoral pseudoaneurysm occurred in 1%, while severe bleeding was observed in only 0.3%. Peri-procedural AMI occurred in 3.6% of cases, transient ischemic attack in 0.4%, and post-contrast acute kidney injury in 1.8%. In-hospital mortality remained low at 1.2%, even in the presence of ACS and a high prevalence of associated comorbidities.

These results underscore the technical proficiency of the tertiary cardiology hospital team, which handles a high volume of procedures. They demonstrate a preference for radial access, the adoption of nephroprotection strategies employing low/isoosmolarity contrasts in small volumes, systematic peri-procedural hydration for patients with a clearance < 60 ml/kg/min, and the universal utilization of new-generation DES.

These findings diverge from those presented by other researchers, indicating elevated mortality rates, particularly in ACS. A meta-analysis encompassing post-ACS studies unveils an approximate 5% mortality rate.^19^ The poorer outcomes could be linked to disparities in access to healthcare services, delayed diagnosis, and inadequate treatment. Studies also indicate that women undergo fewer invasive procedures (such as PCI and surgery) and receive fewer medications recommended by guidelines for the treatment of IHD.^20^

In 2015, Bavishi et al. conducted a meta-analysis comprising 48 studies involving 103,895 patients who underwent PCI following ST-segment elevation myocardial infarction. Their analysis of gender differences in mortality revealed that women experienced a higher mortality rate, particularly in the short term, emphasizing the crucial role of prompt therapy in mitigating adverse outcomes.^21^ This underscores the necessity of implementing standardized emergency department protocols and providing ongoing medical education to address the substantial disparities in the approach to male and female patients, particularly in unstable scenarios.

The predictors of hospital complications in our study align with those documented in the literature. Advanced age, the presence of CKD, and unsuccessful PCI are commonly linked to a poorer prognosis (Table 6).

Advanced age is associated with increased comorbidities, elevated risk of clinical complications, and more complex coronary anatomy. CKD and unsuccessful PCI were identified as predictors of in-hospital MACCE, contributing to adverse early clinical outcomes with a heightened risk of dialysis, cardiac decompensation, angina recurrence, and prolonged hospital stays. Mazurek et al. demonstrated that PCI failure is an independent risk factor for increased mortality in ACS.^22^ Barbash et al. supported this finding, additionally revealing that female gender independently predicts procedure failure in ST-elevation myocardial infarction (OR 1.54; 95% CI [1.01-2.38]).^23^ The predictors of MACCE during the 2-year follow-up were admission for ACS and the presence of in-hospital events.

In the 576-day midterm follow-up, 15% of our patients experienced major cardiac and cerebrovascular outcomes, including cardiac death in 3.5% and recurrent ACS in 8%. The event-free survival rate was 86%. These significant outcomes closely resemble those reported by Mehran et al. after a 2-year follow-up in the Spirit Women study, which randomized women to receive DES with sirolimus or everolimus.^24^

### Limitations

The primary limitation of our study lies in the lack of a comparison with men treated by PCI during the same period, which would be highly desirable to ascertain whether there are indeed differences in immediate and late clinical outcomes post PCI between them.

## Conclusion

In this contemporary study involving 1146 female patients treated by PCI according to guideline-recommended therapies and followed for approximately 2 years, we achieved highly promising in-hospital and mid-term results. It is noteworthy that this research was conducted in a tertiary public hospital, partly during the COVID-19 pandemic, highlighting the challenges inherent in this context.
